# Defining Diffuse Large B-Cell Lymphoma Immunotypes by CD8+ T Cells and Natural Killer Cells

**DOI:** 10.1155/2022/3168172

**Published:** 2022-02-21

**Authors:** Jing Qi, Lu Xu, Dongping Huang, Hesheng He, Junping Yao, Jing Zhang, Youhai Xu, Li Yang

**Affiliations:** ^1^Department of Hematology, The First Affiliated Hospital of Wannan Medical College, Wuhu 241001, Anhui, China; ^2^Department of Hematology, The First Affiliated Hospital of Hainan Medical University, Haikou 570102, Hainan, China; ^3^Department of Hematology, Shanghai Tongren Hospital, Shanghai 200050, China

## Abstract

**Background:**

There is a poor prognosis for diffuse large B-cell lymphoma (DLBCL), one of the most common types of non-Hodgkin lymphoma (NHL). Through gene expression profiles, this study intends to reveal potential subtypes among patients with DLBCL by evaluating their prognostic impact on immune cells.

**Methods:**

Immune subtypes were developed based on CD8+ T cells and natural killer cells calculated from gene expression profiles. The comparison of prognoses and enriched pathways was made between immune subtypes. Following this validation step, samples from the independent data set were analyzed to determine the correlation between immune subtype and prognosis and immune checkpoint blockade (ICB) response. To provide a model to predict the DLBCL immune subtypes, machine learning methods were used. The virtual screening and molecular docking were adopted to identify small molecules to target the immune subtype biomarkers.

**Results:**

A training data set containing 432 DLBCL samples from five data sets and a testing dataset containing 420 DLBCL samples from GSE10846 were used to develop and validate immune subtypes. There were two novel immune subtypes identified in this study: an inflamed subtype (IS) and a noninflamed subtype (NIS). When compared with NIS, IS was associated with higher levels of immune cells and a better prognosis for immunotherapy. Based on the random forest algorithm, a robust machine learning model has been established by 12 hub genes, and the area under the curve (AUC) value is 0.948. Three small molecules were selected to target NIS biomarkers, including VGF, RAD54L, and FKBP8.

**Conclusion:**

This study assessed immune cells as prognostic factors in DLBCL, constructed an immune subtype that could be used to identify patients who would benefit from ICB, and constructed a model to predict the immune subtype.

## 1. Introduction

DLBCL, responsible for nearly 40% of non-Hodgkin lymphoma, is a hematological cancer of B cells [1, 2]. Data on the global epidemiology of DLBCL are scarce, but it is estimated that 7 out of 100,000 people in America suffer from this disease [3]. For patients with DLBCL, chemotherapy agents are the first treatment choice [1]. Although about 65% of DLBCL patients could survive longer than 5 years [4], more than 30% of DLBCL patients still suffer from relapse and ineffective chemotherapy agents [5]. Considering that there are limited treatment options [6], key biomarkers and therapeutic targets are urgently needed.

Recent studies have revealed that the tumor microenvironment (TME) plays a critical role in tumor initiation/progression and response to therapies [7]. Among the components of TME are tumor cells, stromal cells, extracellular matrix, and immune cells such as T cells [8]. As DLBCL is a result of abnormal B-cell development, its malignant cells may also contribute to the dysregulated TME by altering those cytokines that normally control proliferation [9]. As an example, about 30% of DLBCL samples exhibited the loss of HLA-I and CD58 on the surface, which are crucial in the recognition of malignant cells by T cells and natural killer cells [10]. The analysis of TME could explore the relationships of its components with prognosis, making treatment planning in DLBCL more personalized.

PD-L1-positive malignant cells can suppress immune surveillance through a variety of mechanisms, one of which involves the decreased T-cell activity by the PD-1/PD-L1 pathway [11]. Compared with the PD-1-negative subgroup, the DLBCL subgroup with PD-L1+ has an unfavorable prognosis and a reduced overall survival (OS) [12]. An evaluation of the efficacy of pembrolizumab (PD-1 antibody) in combination with R-CHOP in untreated patients with DLBCL demonstrated a 90% overall response rate and a 77% complete response rate. [13]. An additional study revealed that out of five relapsed DLBCL patients, two of them achieved complete remissions through anti-PD-1 therapy and one of them achieved partial remission through anti-PD-1 therapy [14]. There are currently several monoclonal antibodies being developed and evaluated for the treatment of DLBCL that target the PD-1/PD-L1 pathway [5].

Using 432 samples of DLBCL collected from five data sets in the current study, we identified two immune subtypes. In the training and testing data sets, we have analyzed the association between immune subtypes and prognosis, immune cells, and immune pathways. Using the random forest algorithm, 12 genes were selected for the construction of the machine learning model to predict immune subtypes for patients with DLBCL. The machine learning model was further tested through the use of an independent data set. We constructed a 12-gene panel to predict the prognosis of DLBCL patients and validated the prediction using a validation data set of DLBCL patients.

## 2. Materials and Methods

### 2.1. Data Collection and Identification of Immune Subtypes

The training set consisted of 432 DLBCL samples and GSE11318 (*N* = 37) [15], GSE21846 (*N* = 29) [16], GSE23501 (*N* = 69) [17], GSE32918 (*N* = 249) [18], and TCGA-DLBCL (*N* = 48). For the validation of immune subtypes, the 420 DLBCL samples from GSE10846 were chosen as the data set to use for testing [19]. A summary of the demographic data of these DLBCL samples is listed in Table 1. The effect of immune subtypes on immune checkpoint blockade (ICB) response was studied using 65 tumor samples from GSE35640 [20]. All of these samples were retained, including the RNA expression matrix, clinical parameters, and survival data. The mutations of DLBCL samples from TCGA-DLBCL were downloaded from the R package “TCGAmutations”. Tumor mutational burden (TMB) was calculated by dividing the number of nonsynonymous mutations by 38, where 38 is the estimate of the exome size. By using the “GSVA” package, the proportion of 28 types of immune cells was obtained by the expression matrix [21]. According to the values of natural killer cells as well as CD8 T cells, immune subtypes can be determined.

### 2.2. Differentially Expressed Gene (DEG) Identification and Gene Set Enrichment Analysis (GSEA)

We determined the log2FoldChange (FC) values between the inflamed subtype (IS) and noninflamed subtype (NIS) in each training data set using the R language package “limma” [22]. Then, according to *p* value < 0.05 and |log2FoldChange| > 0.5 as the threshold, we used the “RobustRankAggreg” package to find the common and robust DEGs between NIS and IS samples [23]. The package RobustRankAggreg (RRA) could detect genes and proteins that rank consistently better than expected. It could also calculate a significance score for each gene/protein. This method was found to be robust to outliers, noise, and errors [23]. This method was also extensively investigated and used for selecting DEGs in previous articles [24, 25]. In addition, we performed the enrichment analysis according to the log2FoldChange value of robust DEGs. The parameters of GSEA analysis were set as “minSize = 1”, “maxSize = 1000”, and “nperm = 500”.

### 2.3. Gene Selection by Cox Regression Analysis and Random Forest

A univariate Cox regression analysis was performed on the expression profiles of robust DEGs to identify prognostic genes, and immune subtype-related biomarkers were selected from robust DEGs by a cut-off of *p* value  < 0.05 in the univariate Cox regression analysis. Random forest was trained in each training dataset to calculate the importance value of each gene. The top 5 downregulated genes and top 5 upregulated genes with the highest mean value of importance were selected.

### 2.4. The Construction of the Immune Subtype Classifier

Three machine learning algorithms were used in this study, including random forest (RF), support vector machine (SVM), and artificial neural network (ANN). An automatic tuning process was used to adjust the parameters in a 5-fold cross-validation loop. In each loop, parameter values were chosen using a random search with 15 iterations, and model performance was assessed. The best classifier was selected by the area under the curve (AUC) value from 5-fold cross-validation. The prediction performance was further evaluated by the AUC value from the testing data set.

### 2.5. Statistical Analyses

R language software was used to analyze the data. The difference in continuous data across groups was analyzed using the *t*-test. The Kaplan–Meier (KM) and log-rank analyses were used to analyze survival curves. In this study, *p* value < 0.05 was considered to be statistically significant.

### 2.6. Molecular Docking

Virtual screening and molecular docking were computational methods to identify potential small molecules that could target proteins [26]. A list of 1604 small-molecule drugs approved by the FDA was selected and downloaded from the ZINC15 database [27]. The protein structures of three selected proteins (*VGF*, *RAD54L*, and *FKBP8*) were obtained from AlphaFold [28]. AutoDock Vina, a virtual screening software, was used to select the small molecule with the lowest binding energy with the protein [29]. Then, AutoDock, a semiflexible molecular docking software, was used to identify the binding pose of the selected small molecule with the protein [30]. After molecular docking, PyMOL software was used to visualize the binding structures of small molecules and proteins after docking.

## 3. Results

### 3.1. Identification of Immune Subtypes

**Figure 1:**
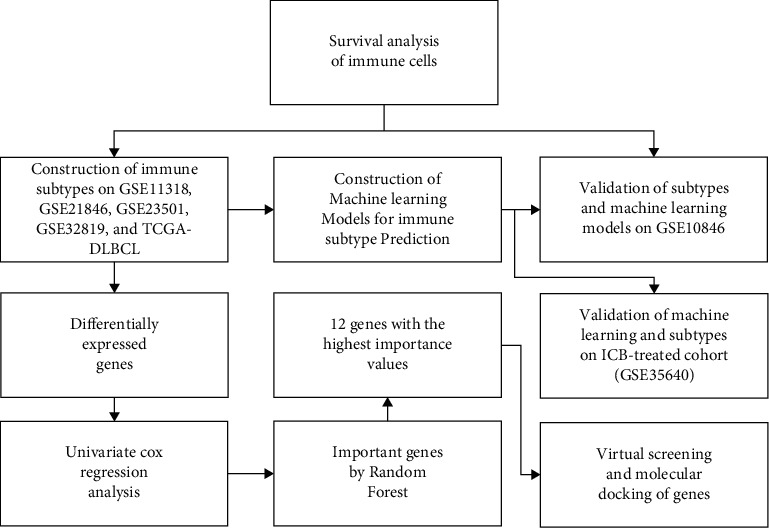
The flowchart of this study.

**Figure 2:**
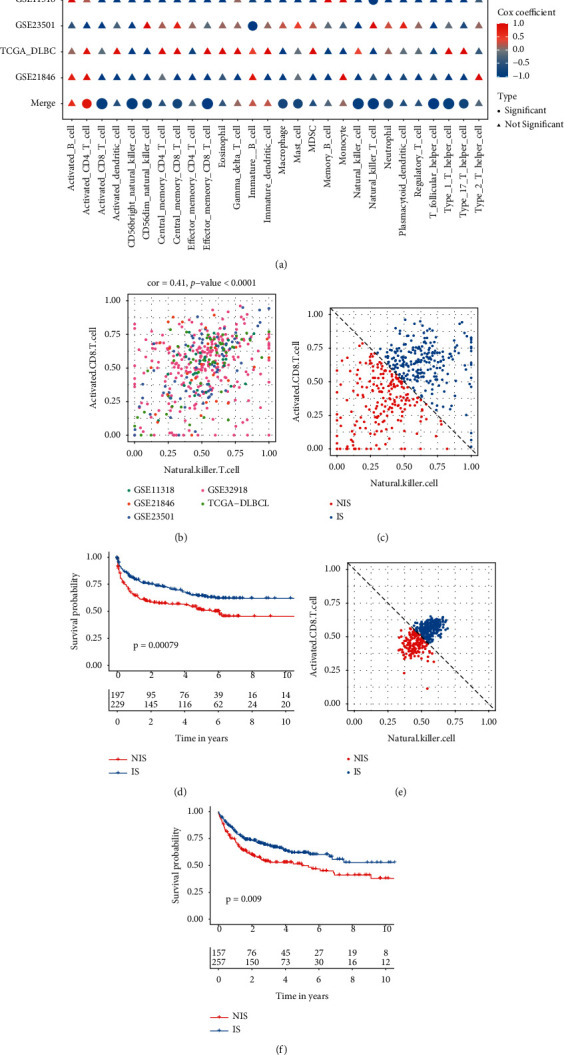
Identification of immune subtypes in the diffuse large B-cell lymphoma (DLBCL) cohorts. (a) The calculated hazard ratios and *p* values of immune cells among DLBCL training cohorts (GSE11318, GSE21846, GSE23501, GSE32819, TCGA-DLBCL, and their merged cohort). (b) The correlation value of natural killer cells and activated CD8 T cells in the merged cohort. (c) In the training data sets, the samples were divided into inflamed subtype (IS) and noninflamed subtype (NIS) based on the diagonal line. IS had higher levels of natural killer cells and activated CD8 T cells. (d) Kaplan–Meier curves of OS for patients in the IS and NIS in the training data set. (e) In the testing data set (GSE10846), the samples were divided into the IS and NIS based on the diagonal line. The IS had higher levels of natural killer cells and activated CD8 T cells. (f) Kaplan–Meier curves of OS for patients in IS and NIS in the testing dataset.

The study flow diagram is shown in Figure 1. In GSE32918, 12 immune cell types were protective (Cox coefficient < 0) and 2 immune cells were hazardous (Cox coefficient > 0, Figure 2(a)). In GSE11318, natural killer T cells were found to be significantly positively related to prognosis. In GSE23501, immature B cells were found to be significantly positively related to prognosis. In TCGA-DLBCL and GSE21846, none of the immune cells had a significant impact on prognosis. The immune cell data from different data sets were then combined. In the combined data set, activated CD8 T cells, CD56 bright natural killer cells, effector memory CD8 T cells, natural killer cells, natural killer T cells, T follicular helper cells, and type 1 T helper cells had a lower *p* value (*p* value <0.01) with prognosis.

As two cytotoxic effector cells of the immune system, activated CD8 T cells and natural killer cells were chosen because they have been implicated in cancer immunotherapy. An association between the number of natural killer cells and the number of activated CD8 T cells was significant in the combined data set (correlation coefficient: 0.41; *p* value < 0.0001; Figure 2(b)). Using coordinate axes and diagonal, we obtained two stable immune subtypes: the inflamed subtype (IS) and the noninflamed subtype (NIS). Samples from IS had more CD8 T lymphocytes along with higher levels of natural killer cells that were both above the diagonal (Figure 2(c)). In contrast, the NIS samples were below the diagonal and had lower levels of activated CD8 T cells and natural killer cells. Using K-M analysis, we were able to determine the correlation between immune subtypes and survival. The overall survival of patients in NIS was significantly shorter than that of patients in subtype IS (Figure 2(d)). In the testing data set, two immune subtypes were derived by the same method (Figure 2(e)). The NIS and IS were statistically different in terms of overall survival, with IS having a better prognosis and NIS suffering from a worse prognosis (Figure 2(f)). Comparing the percentage of pathological stages between the two immune subtype groups is not significant (*p* value = 0.145, Supplementary Table 1).

### 3.2. Comparison of Immune Cells and Immune Function among the Immune Subtypes

**Figure 3:**
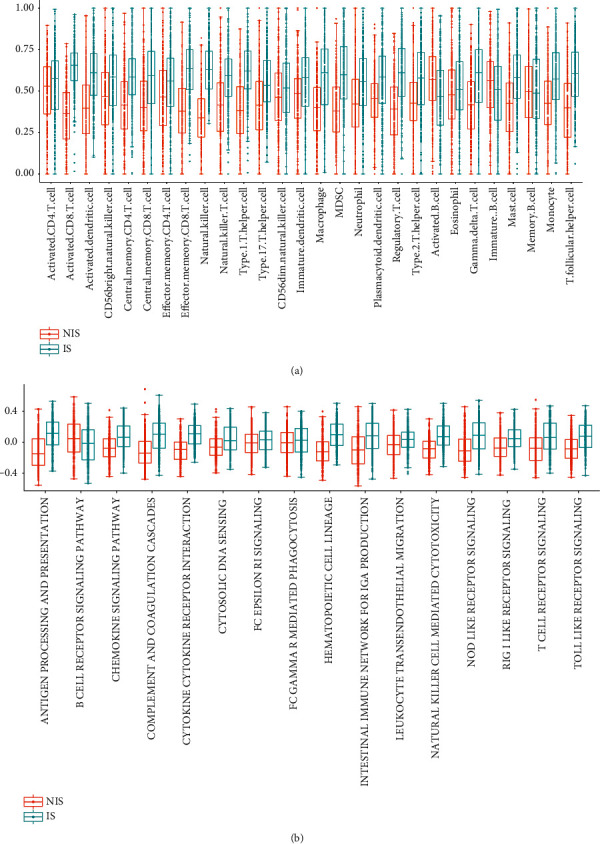
The merged training cohort showed heterogeneity of immune infiltration among noninflamed subtype (NIS) and inflamed subtype (IS). (a) Higher abundance of immune cells such as activated CD4+ T cells, activated CD8+ T cells, and natural killer cells were observed in the IS, while the higher abundance of B cell types containing activated B cells (*p* value < 0.01), immature B cells (*p* value = 0.01), and memory B cells (*p* value = 0.63) were observed in the NIS. (b) For most types of the immune process, they were higher in the IS. But the B cell receptor signaling process was higher in the NIS (*p* value < 0.01).

The combined training data set indicated that the IS was more likely to show infiltration of most types of immune cells, such as T cells in the TME compared with the NIS (Figures 3(a)). However, B cells containing activated B cells (*p* value < 0.01), immature B cells (*p* value = 0.01), and memory B cells (*p* value = 0.63) were higher in the NIS. For a majority of immune functions, they were enriched in the IS (Figures 3(b)). But the B cell receptor signaling was higher in the NIS (*p* value <0.01), which is consistent with the results of immune cell infiltration. In the testing data set (GSE10846), the same results were observed. For example, greater levels of most immune cells (Supplementary Figure 1(a)) and immune functions (Supplementary Figure 1(b)) were found in the IS. But the NIS expressed more B cells and B cell receptor signaling than the IS.

Besides, we also calculated the TMB distribution between the two immune subtypes (Supplementary Figure 2). Although the TMB value appears higher in the NIS, the difference in the TMB value between the two immune subtypes was not significantly different. The expression values of PD-1 and PD-L1 were compared between the two immune subtypes in each data set. In Supplementary Figure 3, the PD-1 expression value was found to be significantly higher in the IS than NIS at GSE10846, GSE11318, GSE21846, GSE23501, and GSE32918 (*p* value < 0.05). In Supplementary Figure 4, the PD-L1 expression value was found to be significantly higher in the IS than NIS at GSE10846, GSE23501, GSE32918, and TCGA-DLBCL (*p* value < 0.05).

### 3.3. Identification of DEGs and Enrichment Analyses

In each training data set, the log2FoldChange values for each gene were obtained by using the “limma” package. Thus, the DEGs lists that contained gene names, log2FoldChange values, and *p* values were obtained. However, the potential DEGs that are crucial for DLBCL development will be hugely reduced if the DEGs lists from different data sets are directly merged. Thus, the RRA method was applied to combine the results from the five data sets with minimal bias. 2409 DEGs (1591 upregulated and 818 downregulated) between IS and NIS were calculated by the RRA method. The robust DEGs were plotted in the heatmap according to the log2FoldChange value (Supplementary Figure 5).

To obtain the enriched pathways, the GO-BP, GO-CC, GO-MF, REACTOME, and KEGG enrichment analyses were applied. For GO-BP analysis, the upregulated genes in the NIS were related to metabolic pathways such as “catabolic process”, “organonitrogen-compound-metabolic process”, and “cellular catabolic process” (Supplementary Table 2). The upregulated genes in the IS were associated with immune pathways such as “response-to-external stimulus”, “defense response”, and “positive regulation of adaptive immune response” (Supplementary Table 3). For GO-CC analysis, the upregulated genes in the NIS were related to “cell junction”, “anchoring junction”, and “nucleolus”. The upregulated genes in the IS were associated with “extracellular space” and “extracellular matrix”. For GO-MF analysis, the upregulated genes in the NIS were related to “RNA binding”, “Poly-A-RNA binding”, and “structural molecule activity”. The upregulated genes in the IS were associated with “receptor binding”, “identical protein binding”, “transition metal-ion binding”, and “cytokine activity”. For KEGG analysis, the upregulated genes in the NIS were mainly related to “ribosome”, “hypertrophic-cardiomyopathy-HCM”, and “adherens junction”. The upregulated genes in the IS were associated with immune pathways “natural-killer-cell-mediated cytotoxicity” and “complement and coagulation cascades”. For REACTOME analysis, the upregulated genes in the NIS were related to metabolic pathways. The upregulated genes in the IS were enriched in immune pathways “innate immune system”, “interferon-alpha-beta signaling”, and “chemokine-receptors-bind-chemokines”.

### 3.4. Gene Selection and Construction of the Immune Subtype Classifier

**Figure 4:**
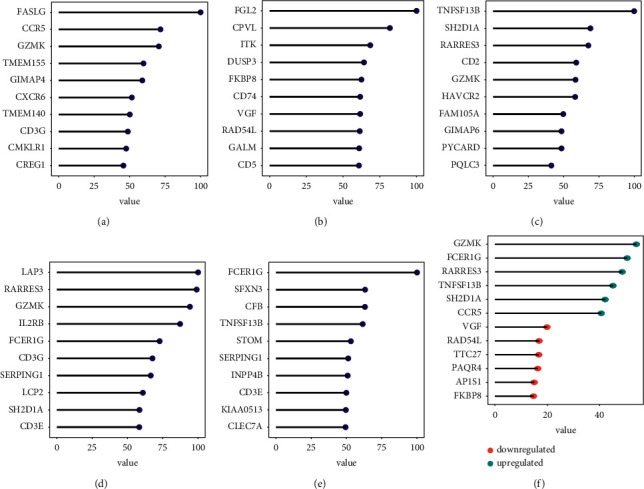
The top 12 most important genes were selected for the construction of machine learning models on the training sets. The top 10 most important genes were selected by a random forest model on the expression data from GSE11318 (a), GSE21846 (b), GSE23501 (c), GSE32918 (d), and TCGA-DLBCL (e). The top 6 upregulated and top 6 downregulated genes in the inflamed subtype (IS) with the highest mean importance value were selected for model construction (f).

A univariate Cox regression analysis was performed to further narrow down the 2409 DEGs. In total, 623 genes were found to be associated with prognosis, 157 of which were associated with poor prognosis and 466 of which were associated with good prognosis. The importance of these 623 genes was evaluated by using the random forest algorithm based on the importance evaluator. The top five most important genes in GSE11318 were *FASLG*, *CCR5*, *GZMK*, *TMEM155*, and *GIMAP4* (Figure 4(a)). *FGL2*, *CPVL*, *ITK*, *DUSP3*, and *FKBP8* comprised the top five genes in GSE21846 (Figure 4(b)). *TNFSF13B*, *SH2D1A*, *RARRES3*, *CD2*, and *GZMK* ranked as the top 5 important genes in GSE23501 (Figure 4(c)). In the analysis of GSE32918, *LAP3*, *RARRES3*, *GZMK*, *IL2RB*, and *FCER1G* were the top five genes involved (Figure 4(d)). The top 5 genes in the TCGA-DLBCL were *FCER1G*, *SFXN3*, *CFB*, *TNFSF13B*, and *STOM* (Figure 4(e)). The top 6 upregulated DEGs with the greatest mean importance value (*GZMK*, *FCER1G*, *RARRES3*, *TNFSF13B*, *SH2D1A*, and *CCR5*) and the top 6 downregulated DEGs with the highest importance value (*VGF*, *RAD54L*, *TTC27*, *PAQR4*, *AP1S1*, and *FKBP8*) were chosen for model creation (Figure 4(f)).

**Figure 5:**
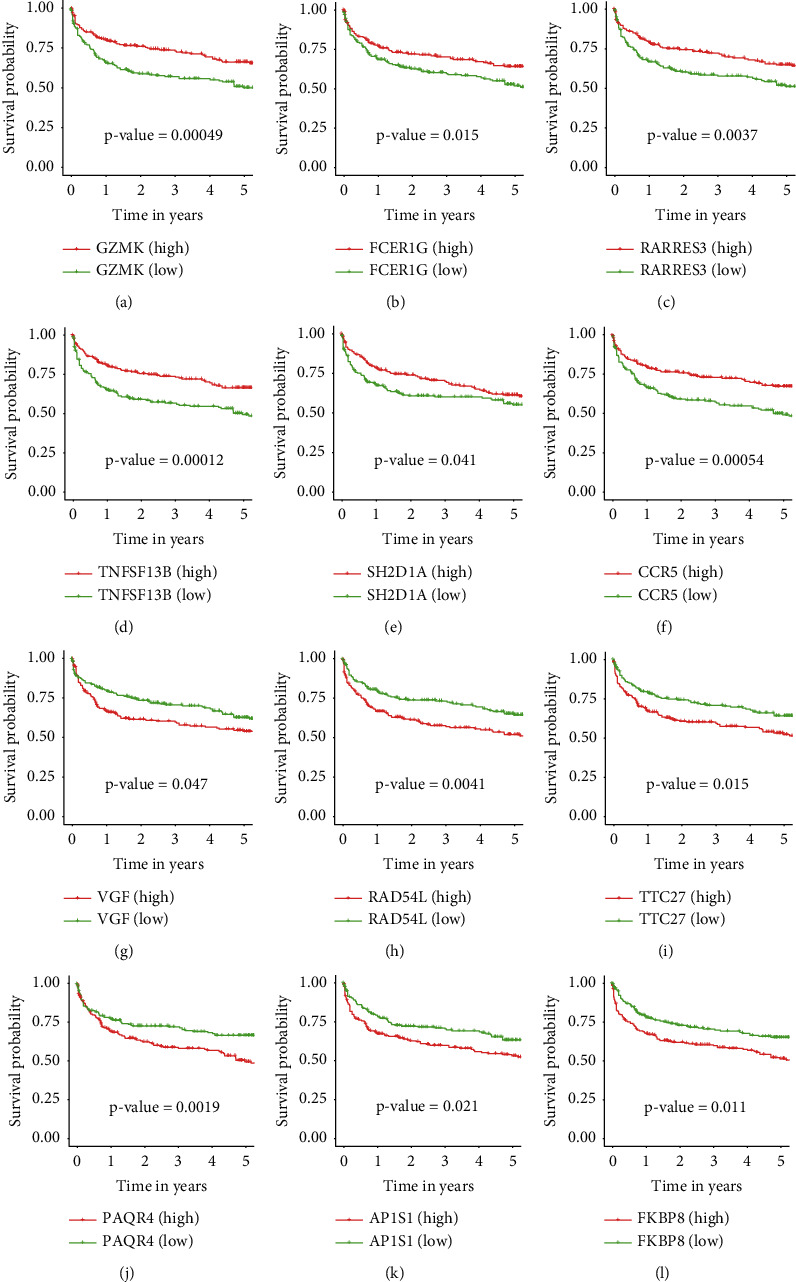
Analysis of the relationship between 12 selected genes and diffuse large B-cell lymphoma (DLBCL) overall survival prognosis based on the Kaplan–Meier plotter in the training set. (a–f) Kaplan–Meier plots of survival analysis of 6 upregulated genes. (g–l) Kaplan–Meier plots of survival analysis of 6 downregulated genes.

The expression values of these 6 upregulated (*GZMK*, *FCER1G*, *RARRES3*, *TNFSF13B*, *SH2D1A*, and *CCR5*) and 6 downregulated DEGs (*VGF*, *RAD54L*, *TTC27*, *PAQR4*, *AP1S1*, and *FKBP8*) in the IS and NIS from the testing data set are shown in Supplementary Figure 6. Furthermore, K-M survival curves were created to analyze the relationships between the expression levels of the 12 genes and OS. In the combined training data set, upregulated genes were associated with a better prognosis, while downregulated genes were associated with a worse prognosis (Figure 5). As shown in Supplementary Figure 7, OS curves for *TNFSF13B*, *VGF*, *RAD54L*, and *FKBP8* in the testing data set were noticeably different. The correlations of 12 genes with immune cells are plotted in Supplementary Figure 8. *GZMK*, *FCER1G*, *RARRES3*, *TNFSF13B*, *SH2D1A*, and *CCR5* were found to be positively correlated with immune cells such as T cells, and these genes were negatively correlated with B cells. In contrast, *VGF*, *RAD54L*, *TTC27*, *PAQR4*, *AP1S1*, and *FKBP8* were found to be negatively correlated with immune cells such as T cells, and these genes were positively correlated with B cells.

**Figure 6:**
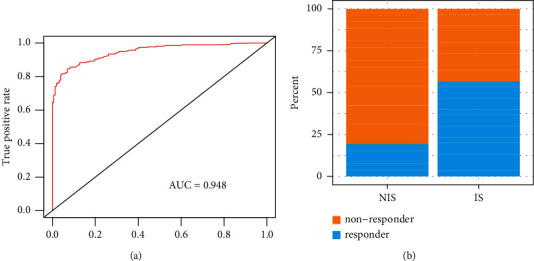
The validation of the random forest model by independent testing data sets. (a) The area under the curve (AUC) value of the constructed random forest model on the testing data set (GSE10486). (b) The validation of correlation of the predicted immune subtype with immune checkpoint blockade (ICB) drug response.

As shown in Table 2, random forest (RF) yields an AUC of 0.908, support vector machine (SVM) yields 0.907, and artificial neural network (ANN) yields 0.898. The random forest was selected since it had the highest AUC value. The constructed random forest model reached an AUC of 0.948 in the testing data set (Figure 6(a)). Based on the expression data of GSE35640, the immune subtype of cancer patients who received the treatment of immunotherapy was predicted. We found that the response rate to immunotherapy for the IS was higher than the NIS (0.57 vs 0.19) (Figure 6(b)). Consequently, the constructed model can serve as a useful tool to select patients who are likely to benefit from immunotherapy.

### 3.5. Virtual Screening and Molecular Docking Analysis

**Figure 7:**
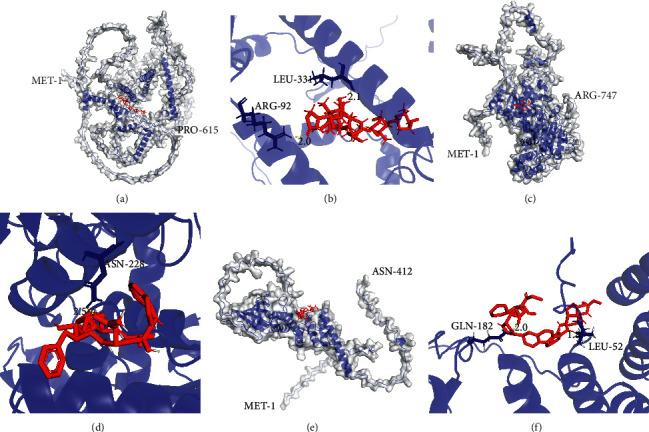
3D structures of proteins (blue color) with small molecules (red color). (a-b) Schematic of intermolecular interaction of the binding mode of VGF protein with ZINC242548690. (c-d) Schematic of intermolecular interaction of the binding mode of RAD54L protein with ZINC29416466. (e-f) Schematic of intermolecular interaction of the binding mode of FKBP8 protein with ZINC203686879.

According to the virtual screening results from AutoDock Vina, the small molecules with the lowest free energy for each protein were selected. ZINC242548690, ZINC29416466, and ZINC203686879 were selected to target VGF, RAD54L, and FKBP8, respectively. Next, AutoDock and PyMOL were used to dock and visualize small molecules and proteins. The binding poses of protein-molecule complexes were ranked by the binding free energy. The 3D images of binding poses with the lowest energy are shown in Figure 7. In addition, 2, 1, and 2 H-bonds were found among these three protein-molecule complexes.

## 4. Discussion

The DLBCL is an aggressive, clinically, and genetically heterogeneous disease. From the origin cell, it can be further classified into transcriptionally defined activated B cells (ABCs) and germinal center B cells (GCBs) [31]. DLBCL subtypes have been or will be taken into consideration for the treatment of DLBCL. A prior study, for example, performed a thorough genetic analysis to identify five distinct DLBCL molecular subtypes [31]. The purpose of this study was to analyze the immune subtypes of DLBCL based on specific immune cells and to evaluate the reliability of the findings. Based on CT8 T cells and natural killer cells, two distinct subtypes were identified in our study: the inflamed subtype (IS) and the noninflamed subtype (NIS). The IS was associated with immune cells such as T cells. But the NIS was associated with B cells and B-cell-related pathways. Furthermore, survival analysis showed that IS had a better prognosis than the NIS.

Several studies have revealed that the TME plays a significant role in the ICB therapy response rate [32]. In this work, supervised machine learning approaches were used to build models that could predict DLBCL patients' immune subtypes. The impact of immune subtypes on ICB responsiveness was then proven. We discovered that the IS had a greater response rate to immunotherapy than the NIS (0.57 vs 0.19). As a result, our machine learning model may provide a method for selecting DLBCL patients for ICB therapy.

For the NIS patients, six genes (*VGF*, *RAD54L*, *TTC27*, *PAQR4*, *AP1S1*, and *FKBP8*) were selected as the subtype biomarkers. Since three of them (*VGF*, *RAD54L*, and *FKBP8*) showed notable differences in prognosis from the testing data set, they were selected as the target proteins to identify the potential small-molecule drugs. Based on the virtual screening and molecular docking analysis, three small molecules were finally selected as the novel therapeutic drugs for NIS patients. The NIS might be sensitive to three selected small molecules: ZINC242548690, ZINC29416466, and ZINC203686879. ZINC242548690 (digoxin) is a cardiac glycoside, but many studies suggested it could increase the effect of anticancer therapy [33]. ZINC29416466 (saquinavir) is an available human immunodeficiency virus protease inhibitor and could inhibit proteasome activity in mammalian cells as well as act on the HIV-I protease [34]. Saquinavir was found to induce apoptosis in human cancer cells and could become a new class of cytotoxic chemotherapy drugs [34, 35]. ZINC203686879 (velpatasvir) is one of the hepatitis virus inhibitors [36], and studies are needed to validate the effect of velpatasvir on tumor cells. This study aims to provide novel therapies to the need for personalized and precise treatment for DLBCL patients.

There are certain limitations to our research. *In vitro* and *in vivo* testing should be done on the effects of ZINC242548690, ZINC29416466, and ZINC203686879 on DLBCL tumor development. Additionally, the immune cells in this study were solely predicted by the R GSVA package. There will be a more precise evaluation of immune cells if experiments or multiple bioinformatics methods can be conducted.

## 5. Conclusion

Our study discovered the correlations of immune cells with prognosis. Based on the CD8 T cell and natural killer cells, DLBCL samples were divided into NIS and IS. Multiple cohorts evaluated and confirmed the associations of immune subtypes with prognosis and ICB therapy responsiveness. In conclusion, we constructed an accurate and robust machine learning model that may facilitate the prediction of immune subtypes and DLBCL patient selection for ICB treatment.

## Figures and Tables

**Table 1:** Additional clinical information DLBCL patients from GSE11318, GSE21846, GSE23501, GSE32918, and TCGA-DLBCL.

Clinical information	GSE11318 (*N* = 37)	GSE21846 (*N* = 29)	GSE23501 (*N* = 69)	GSE32918 (*N* = 249)	TCGA-DLBCL (*N* = 48)	GSE10846 (*N* = 420)
Gender	Male (*N* = 20)	Not available	Male (*N* = 50)	Male (*N* = 144)	Male (*N* = 22)	Male (*N* = 224)
	Female (*N* = 17)		Female (*N* = 19)	Female (*N* = 105)	Female (*N* = 26)	Female (*N* = 172)
						Not available (*N* = 24)

Median age (years)	Not available	Not available	66	69	57	62
Overall survival status	Not available	Dead (*N* = 25)	Dead (*N* = 13)	Dead (*N* = 137)	Dead (*N* = 5)	Dead (*N* = 165)
		Alive (*N* = 4)	Alive (*N* = 56)	Alive (*N* = 112)	Alive (*N* = 43)	Alive (*N* = 249)
						Not available (*N* = 6)

Median overall survival time (months)	Not available	18	22	48	13	28

**Table 2:** The AUC value results from machine learning models by 5-fold cross-validation in the training data set. The parameter of the machine learning model with the highest value was selected for the prediction model construction.

Type	mtry	C	Decay	ROC	Sens	Spec
Random forest	2	NA	NA	0.908	0.807	0.817
Random forest	3	NA	NA	0.907	0.821	0.805
Random forest	4	NA	NA	0.902	0.807	0.805
Random forest	6	NA	NA	0.901	0.821	0.811
Random forest	5	NA	NA	0.899	0.814	0.799
SVM	NA	0.25	NA	0.907	0.793	0.817
SVM	NA	0.5	NA	0.904	0.8	0.817
SVM	NA	1	NA	0.901	0.771	0.835
SVM	NA	2	NA	0.899	0.8	0.83
SVM	NA	4	NA	0.897	0.807	0.835
ANN	NA	NA	0.9	0.898	0.786	0.83
ANN	NA	NA	0.7	0.898	0.793	0.842
ANN	NA	NA	0.8	0.897	0.793	0.842
ANN	NA	NA	0.6	0.896	0.786	0.836
ANN	NA	NA	0.5	0.895	0.786	0.83

## Data Availability

The data sets were downloaded from the open-access data sets without limitations, and the resources are listed in “Data Collection” and “Identification of Immune Subtypes” of the “Materials and Methods” section.

## Supplementary Materials

Supplementary Materials Supplementary Figure 1. The testing cohort showed heterogeneity of immune infiltration among the NIS and IS. (a) Higher abundance of immune cells such as activated CD4+ T cells, activated CD8+ T cells, and natural killer cells were observed in the IS, while higher abundance of B cell types containing activated B cells (*p* value = 0.02), immature B cells (*p* value = 0.65), and memory B cells (*p* value = 0.39) were higher in the NIS. (b) For most types of the immune process, they were higher in the IS. But the B cell receptor signaling process was not significantly different between the two subtypes (*p* value = 0.53). Supplementary Figure 2. Boxplot distribution of tumor mutational burden (TMB) values between inflamed subtype (NIS) and inflamed subtype (IS) from TCGA-DLBCL. Supplementary Figure 3. Boxplot distribution of expression data of PD-1 between inflamed subtype (NIS) and inflamed subtype (IS) from six data sets. Supplementary Figure 4. Boxplot distribution of expression data of PD-L1 between inflamed subtype (NIS) and inflamed subtype (IS) from six data sets. Supplementary Figure 5. Identification of differentially expressed genes (DEGs) between inflamed subtype (NIS) and inflamed subtype (IS) by expression profiling of TCGA-DLBCL, GSE21846, GSE32918, GSE11318, and GSE23501 data sets. The significantly upregulated and downregulated DEGs were shown in a heatmap by log2FoldChange values. Red represents higher expression and green represents lower expression in IS samples. Supplementary Figure 6. Boxplot distribution of expression data of 12 selected genes and between inflamed subtype (NIS) and inflamed subtype (IS) from the testing set (GSE10846). Supplementary Figure 7. Analysis of the relationship between 12 selected genes and diffuse large B-cell lymphoma (DLBCL) overall survival prognosis based on the Kaplan–Meier plotter in the testing set (GSE10846). (a–f) Kaplan–Meier plots of survival analysis of 6 upregulated genes. (g–l) Kaplan–Meier plots of survival analysis of 6 downregulated genes. Supplementary Figure 8. Correlation analysis of 12 genes with immune cells in GSE10846. Supplementary Table 1. Analysis of the correlation of immune subtype with tumorigenesis-related clinical information, such as stages (GSE10846). Supplementary Table 2. The enriched GO-BP, GO-CC, GO-MF, KEGG, and REACTOME pathways associated with DEGs of the NIS predicted by GSEA analysis. Supplementary Table 3. The enriched GO-BP, GO-CC, GO-MF, KEGG, and REACTOME pathways of the IS predicted by GSEA analysis.
