# Supplementary material for: Defining Diffuse Large B-Cell Lymphoma Immunotypes by CD8+ T Cells and Natural Killer Cells
Source: J Oncol. 2022 Feb 21;2022:3168172. doi: 10.1155/2022/3168172 (PMC8885174; doi:10.1155/2022/3168172)
Supplement: Supplementary Materials — Supplementary Figure 1. The testing cohort showed heterogeneity of immune infiltration among the NIS and IS. (a) Higher abundance of immune cells such as activated CD4+ T cells, activated CD8+ T cells, and natural killer cells were observed in the IS, while higher abundance of B cell types containing activated B cells (p value = 0.02), immature B cells (p value = 0.65), and memory B cells (p value = 0.39) were higher in the NIS. (b) For most types of the immune process, they were higher in the IS. But the B cell receptor signaling process was not significantly different between the two subtypes (p value = 0.53). Supplementary Figure 2. Boxplot distribution of tumor mutational burden (TMB) values between inflamed subtype (NIS) and inflamed subtype (IS) from TCGA-DLBCL. Supplementary Figure 3. Boxplot distribution of expression data of PD-1 between inflamed subtype (NIS) and inflamed subtype (IS) from six data sets. Supplementary Figure 4. Boxplot distribution of expression data of PD-L1 between inflamed subtype (NIS) and inflamed subtype (IS) from six data sets. Supplementary Figure 5. Identification of differentially expressed genes (DEGs) between inflamed subtype (NIS) and inflamed subtype (IS) by expression profiling of TCGA-DLBCL, GSE21846, GSE32918, GSE11318, and GSE23501 data sets. The significantly upregulated and downregulated DEGs were shown in a heatmap by log2FoldChange values. Red represents higher expression and green represents lower expression in IS samples. Supplementary Figure 6. Boxplot distribution of expression data of 12 selected genes and between inflamed subtype (NIS) and inflamed subtype (IS) from the testing set (GSE10846). Supplementary Figure 7. Analysis of the relationship between 12 selected genes and diffuse large B-cell lymphoma (DLBCL) overall survival prognosis based on the Kaplan–Meier plotter in the testing set (GSE10846). (a–f) Kaplan–Meier plots of survival analysis of 6 upregulated genes. (g–l) Kaplan–Meier plots of survival anal [file 3168172.f1.zip › Supplementary Table 2.docx]

| **Type** | **Pathway** | **P-value** | **NES** | **Size** |
| --- | --- | --- | --- | --- |
| GO-BP | CATABOLIC PROCESS | 0.003 | -1.91 | 354 |
| GO-BP | PROTEIN LOCALIZATION | 0.003 | -1.96 | 346 |
| GO-BP | ORGANONITROGEN COMPOUND METABOLIC PROCESS | 0.003 | -2.03 | 334 |
| GO-BP | ESTABLISHMENT OF LOCALIZATION IN CELL | 0.003 | -1.99 | 296 |
| GO-BP | ESTABLISHMENT OF PROTEIN LOCALIZATION | 0.003 | -2.02 | 292 |
| GO-BP | CELLULAR CATABOLIC PROCESS | 0.003 | -2.00 | 278 |
| GO-BP | CELLULAR MACROMOLECULE LOCALIZATION | 0.003 | -2.02 | 245 |
| GO-BP | MACROMOLECULAR COMPLEX ASSEMBLY | 0.026 | -1.35 | 244 |
| GO-BP | ORGANONITROGEN COMPOUND BIOSYNTHETIC PROCESS | 0.003 | -2.19 | 202 |
| GO-BP | MEMBRANE ORGANIZATION | 0.003 | -2.03 | 193 |
| GO-CC | CELL JUNCTION | 0.003 | -1.83 | 210 |
| GO-CC | ANCHORING JUNCTION | 0.003 | -1.85 | 135 |
| GO-CC | NUCLEOLUS | 0.003 | -1.88 | 134 |
| GO-CC | CELL SUBSTRATE JUNCTION | 0.003 | -1.82 | 123 |
| GO-CC | RIBONUCLEOPROTEIN COMPLEX | 0.003 | -2.11 | 114 |
| GO-CC | CYTOSOLIC PART | 0.003 | -2.06 | 70 |
| GO-CC | RIBOSOME | 0.004 | -2.03 | 62 |
| GO-CC | RIBOSOMAL SUBUNIT | 0.004 | -2.02 | 57 |
| GO-CC | CYTOSOLIC RIBOSOME | 0.004 | -1.99 | 50 |
| GO-CC | TRANS GOLGI NETWORK | 0.033 | -1.48 | 42 |
| GO-MF | RNA BINDING | 0.003 | -2.12 | 248 |
| GO-MF | POLA RNA BINDING | 0.003 | -2.02 | 191 |
| GO-MF | STRUCTURAL MOLECULE ACTIVITY | 0.003 | -2.07 | 124 |
| GO-MF | STRUCTURAL CONSTITUENT OF RIBOSOME | 0.0038 | -2.01 | 63 |
| GO-MF | MRNA BINDING | 0.025 | -1.64 | 24 |
| GO-MF | RRNA BINDING | 0.004 | -1.73 | 20 |
| GO-MF | NUCLEOSOME BINDING | 0.044 | -1.60 | 9 |
| GO-MF | MHC PROTEIN COMPLEX BINDING | 0.043 | -1.65 | 8 |
| GO-MF | NITRIC OXIDE SYNTHASE BINDING | 0.029 | -1.64 | 5 |
| GO-MF | KINESIN BINDING | 0.029 | -1.63 | 5 |
| KEGG | RIBOSOME | 0.004 | -1.98 | 49 |
| KEGG | HYPERTROPHIC CARDIOMYOPATHY HCM | 0.004 | -1.58 | 19 |
| KEGG | ADHERENS JUNCTION | 0.014 | -1.57 | 17 |
| KEGG | DILATED CARDIOMYOPATHY | 0.009 | -1.60 | 16 |
| KEGG | VIBRIO CHOLERAE INFECTION | 0.018 | -1.54 | 15 |
| KEGG | ARRHYTHMOGENIC RIGHT VENTRICULAR CARDIOMYOPATHY ARVC | 0.009 | -1.60 | 14 |
| REACTOME | METABOLISM OF PROTEINS | 0.003 | -2.16 | 105 |
| REACTOME | CELL CYCLE | 0.003 | -1.60 | 79 |
| REACTOME | METABOLISM OF RNA | 0.003 | -2.07 | 68 |
| REACTOME | METABOLISM OF MRNA | 0.003 | -2.06 | 65 |
| REACTOME | CELL CYCLE MITOTIC | 0.003 | -1.60 | 63 |
| REACTOME | TRANSLATION | 0.003 | -2.06 | 61 |
| REACTOME | INFLUENZA LIFE CYCLE | 0.004 | -2.02 | 58 |
| REACTOME | SRP DEPENDENT COTRANSLATIONAL PROTEIN TARGETING TO MEMBRANE | 0.003 | -2.02 | 53 |
| REACTOME | INFLUENZA VIRAL RNA TRANSCRIPTION AND REPLICATION | 0.003 | -2.02 | 53 |
| REACTOME | NONSENSE MEDIATED DECAY_ENHANCED BY THE EXON JUNCTION COMPLEX | 0.003 | -2.03 | 53 |

**Supplementary Table 2.** The enriched GO-BP, GO-CC, GO-MF, KEGG, and REACTOME pathways associated with DEGs of the NIS subtype predicted by GSEA analysis.
