# Supplementary material for: Defining Diffuse Large B-Cell Lymphoma Immunotypes by CD8+ T Cells and Natural Killer Cells
Source: J Oncol. 2022 Feb 21;2022:3168172. doi: 10.1155/2022/3168172 (PMC8885174; doi:10.1155/2022/3168172)
Supplement: Supplementary Materials — Supplementary Figure 1. The testing cohort showed heterogeneity of immune infiltration among the NIS and IS. (a) Higher abundance of immune cells such as activated CD4+ T cells, activated CD8+ T cells, and natural killer cells were observed in the IS, while higher abundance of B cell types containing activated B cells (p value = 0.02), immature B cells (p value = 0.65), and memory B cells (p value = 0.39) were higher in the NIS. (b) For most types of the immune process, they were higher in the IS. But the B cell receptor signaling process was not significantly different between the two subtypes (p value = 0.53). Supplementary Figure 2. Boxplot distribution of tumor mutational burden (TMB) values between inflamed subtype (NIS) and inflamed subtype (IS) from TCGA-DLBCL. Supplementary Figure 3. Boxplot distribution of expression data of PD-1 between inflamed subtype (NIS) and inflamed subtype (IS) from six data sets. Supplementary Figure 4. Boxplot distribution of expression data of PD-L1 between inflamed subtype (NIS) and inflamed subtype (IS) from six data sets. Supplementary Figure 5. Identification of differentially expressed genes (DEGs) between inflamed subtype (NIS) and inflamed subtype (IS) by expression profiling of TCGA-DLBCL, GSE21846, GSE32918, GSE11318, and GSE23501 data sets. The significantly upregulated and downregulated DEGs were shown in a heatmap by log2FoldChange values. Red represents higher expression and green represents lower expression in IS samples. Supplementary Figure 6. Boxplot distribution of expression data of 12 selected genes and between inflamed subtype (NIS) and inflamed subtype (IS) from the testing set (GSE10846). Supplementary Figure 7. Analysis of the relationship between 12 selected genes and diffuse large B-cell lymphoma (DLBCL) overall survival prognosis based on the Kaplan–Meier plotter in the testing set (GSE10846). (a–f) Kaplan–Meier plots of survival analysis of 6 upregulated genes. (g–l) Kaplan–Meier plots of survival anal [file 3168172.f1.zip › Supplementary Table 3.docx]

| **Type** | **Pathway** | **p-value** | **NES** | **Size** |
| --- | --- | --- | --- | --- |
| GO-BP | RESPONSE TO EXTERNAL STIMULUS | 0.034 | 1.39 | 403 |
| GO-BP | DEFENSE RESPONSE | 0.016 | 1.43 | 383 |
| GO-BP | CHEMICAL HOMEOSTASIS | 0.012 | 1.69 | 182 |
| GO-BP | ION TRANSPORT | 0.004 | 1.71 | 179 |
| GO-BP | CELLULAR HOMEOSTASIS | 0.008 | 1.75 | 147 |
| GO-BP | CELLULAR CHEMICAL HOMEOSTASIS | 0.008 | 1.77 | 121 |
| GO-BP | ION HOMEOSTASIS | 0.004 | 1.80 | 118 |
| GO-BP | CATION TRANSPORT | 0.004 | 1.79 | 113 |
| GO-BP | CELL CELL SIGNALING | 0.040 | 1.66 | 105 |
| GO-BP | METAL ION TRANSPORT | 0.007 | 1.80 | 87 |
| GO-BP | POSITIVE REGULATION OF ADAPTIVE IMMUNE RESPONSE | 0.047 | 1.78 | 36 |
| GO-CC | EXTRACELLULAR SPACE | 0.004 | 1.56 | 302 |
| GO-CC | EXTRACELLULAR MATRIX | 0.032 | 1.64 | 74 |
| GO-CC | EARLY ENDOSOME | 0.027 | 1.72 | 68 |
| GO-CC | VACUOLAR LUMEN | 0.034 | 1.60 | 52 |
| GO-CC | ENDOPLASMIC RETICULUM LUMEN | 0.034 | 1.59 | 44 |
| GO-CC | PHAGOCYTIC VESICLE | 0.014 | 1.65 | 31 |
| GO-CC | EARLY ENDOSOME MEMBRANE | 0.010 | 1.78 | 28 |
| GO-CC | PHAGOCYTIC VESICLE MEMBRANE | 0.017 | 1.70 | 27 |
| GO-CC | COLLAGEN TRIMER | 0.006 | 1.82 | 16 |
| GO-CC | PROTEIN LIPID COMPLEX | 0.011 | 1.79 | 12 |
| GO-MF | RECEPTOR BINDING | 0.018 | 1.50 | 297 |
| GO-MF | IDENTICAL PROTEIN BINDING | 0.033 | 1.55 | 235 |
| GO-MF | TRANSITION METAL ION BINDING | 0.039 | 1.63 | 174 |
| GO-MF | CYTOKINE ACTIVITY | 0.044 | 1.68 | 55 |
| GO-MF | G PROTEIN COUPLED RECEPTOR BINDING | 0.036 | 1.72 | 52 |
| GO-MF | SERINE HYDROLASE ACTIVITY | 0.036 | 1.72 | 41 |
| GO-MF | ORGANIC ACID BINDING | 0.040 | 1.62 | 35 |
| GO-MF | CHEMOKINE RECEPTOR BINDING | 0.035 | 1.70 | 27 |
| GO-MF | CYSTEINE TYPE PEPTIDASE ACTIVITY | 0.034 | 1.64 | 26 |
| GO-MF | GLYCOPROTEIN BINDING | 0.034 | 1.752 | 26 |
| KEGG | LYSOSOME | 0.047 | 1.61 | 60 |
| KEGG | NATURAL KILLER CELL MEDIATED CYTOTOXICITY | 0.037 | 1.63 | 44 |
| KEGG | ENDOCYTOSIS | 0.046 | 1.62 | 35 |
| KEGG | TOLL LIKE RECEPTOR SIGNALING PATHWAY | 0.046 | 1.55 | 32 |
| KEGG | COMPLEMENT AND COAGULATION CASCADES | 0.021 | 1.74 | 21 |
| KEGG | PRION DISEASES | 0.003 | 1.77 | 10 |
| REACTOME | METABOLISM OF LIPIDS AND LIPOPROTEINS | 0.044 | 1.54 | 83 |
| REACTOME | INNATE IMMUNE SYSTEM | 0.03 | 1.58 | 81 |
| REACTOME | TRANSMEMBRANE TRANSPORT OF SMALL_MOLECULES | 0.003 | 1.90 | 69 |
| REACTOME | CLASS A1 RHODOPSIN LIKE RECEPTORS | 0.023 | 1.61 | 57 |
| REACTOME | CLASS I MHC MEDIATED ANTIGEN PROCESSING PRESENTATION | 0.034 | 1.59 | 47 |
| REACTOME | IMMUNOREGULATORY INTERACTIONS BETWEEN A LYMPHOID AND A NON LYMPHOID CELL | 0.016 | 1.68 | 40 |
| REACTOME | PEPTIDE LIGAND BINDING RECEPTORS | 0.031 | 1.64 | 39 |
| REACTOME | INTERFERON ALPHA BETA SIGNALING | 0.026 | 1.64 | 35 |
| REACTOME | CHEMOKINE RECEPTORS BIND CHEMOKINES | 0.038 | 1.69 | 26 |
| REACTOME | MEMBRANE_TRAFFICKING | 0.007 | 1.84 | 22 |

**Supplementary Table 3.** The enriched GO-BP, GO-CC, GO-MF, KEGG, and REACTOME pathways of the IS subtype predicted by GSEA analysis.
