# Supplementary figures and images for: Defining Diffuse Large B-Cell Lymphoma Immunotypes by CD8+ T Cells and Natural Killer Cells
Source: J Oncol. 2022 Feb 21;2022:3168172. doi: 10.1155/2022/3168172 (PMC8885174; doi:10.1155/2022/3168172)

**A**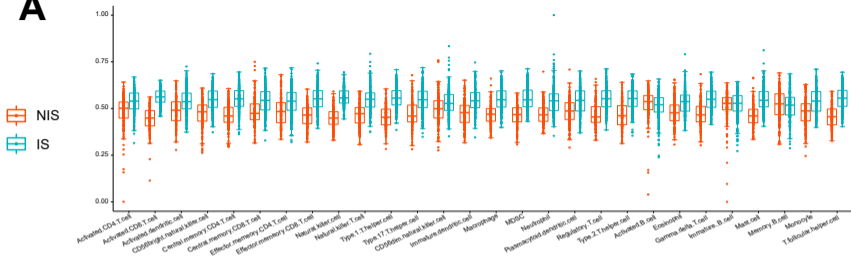**B**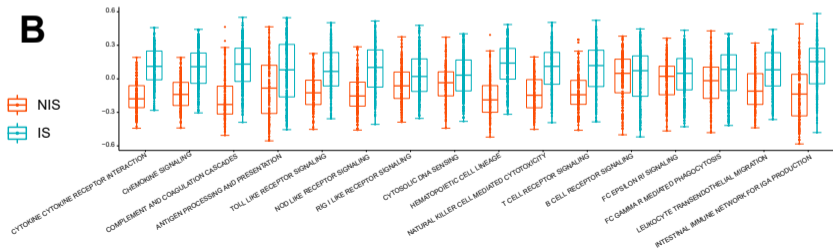

Supplement: Supplementary Materials — Supplementary Figure 1. The testing cohort showed heterogeneity of immune infiltration among the NIS and IS. (a) Higher abundance of immune cells such as activated CD4+ T cells, activated CD8+ T cells, and natural killer cells were observed in the IS, while higher abundance of B cell types containing activated B cells (p value = 0.02), immature B cells (p value = 0.65), and memory B cells (p value = 0.39) were higher in the NIS. (b) For most types of the immune process, they were higher in the IS. But the B cell receptor signaling process was not significantly different between the two subtypes (p value = 0.53). Supplementary Figure 2. Boxplot distribution of tumor mutational burden (TMB) values between inflamed subtype (NIS) and inflamed subtype (IS) from TCGA-DLBCL. Supplementary Figure 3. Boxplot distribution of expression data of PD-1 between inflamed subtype (NIS) and inflamed subtype (IS) from six data sets. Supplementary Figure 4. Boxplot distribution of expression data of PD-L1 between inflamed subtype (NIS) and inflamed subtype (IS) from six data sets. Supplementary Figure 5. Identification of differentially expressed genes (DEGs) between inflamed subtype (NIS) and inflamed subtype (IS) by expression profiling of TCGA-DLBCL, GSE21846, GSE32918, GSE11318, and GSE23501 data sets. The significantly upregulated and downregulated DEGs were shown in a heatmap by log2FoldChange values. Red represents higher expression and green represents lower expression in IS samples. Supplementary Figure 6. Boxplot distribution of expression data of 12 selected genes and between inflamed subtype (NIS) and inflamed subtype (IS) from the testing set (GSE10846). Supplementary Figure 7. Analysis of the relationship between 12 selected genes and diffuse large B-cell lymphoma (DLBCL) overall survival prognosis based on the Kaplan–Meier plotter in the testing set (GSE10846). (a–f) Kaplan–Meier plots of survival analysis of 6 upregulated genes. (g–l) Kaplan–Meier plots of survival anal [file 3168172.f1.zip › supplementary Figure 1.pdf]

IS

NIS

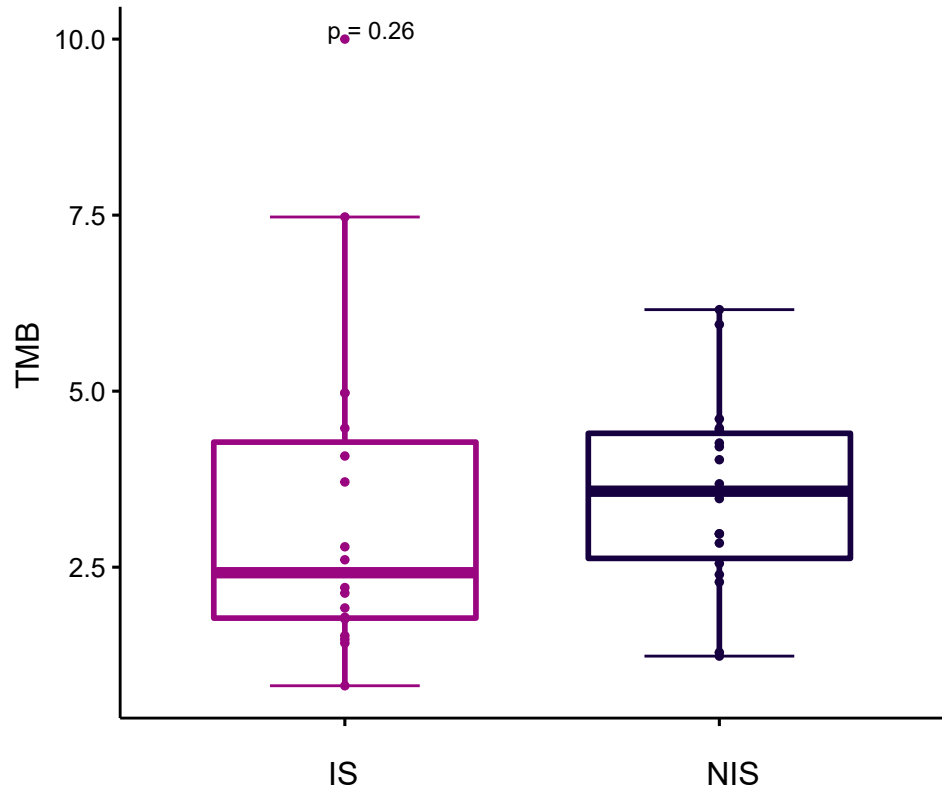

Supplement: Supplementary Materials — Supplementary Figure 1. The testing cohort showed heterogeneity of immune infiltration among the NIS and IS. (a) Higher abundance of immune cells such as activated CD4+ T cells, activated CD8+ T cells, and natural killer cells were observed in the IS, while higher abundance of B cell types containing activated B cells (p value = 0.02), immature B cells (p value = 0.65), and memory B cells (p value = 0.39) were higher in the NIS. (b) For most types of the immune process, they were higher in the IS. But the B cell receptor signaling process was not significantly different between the two subtypes (p value = 0.53). Supplementary Figure 2. Boxplot distribution of tumor mutational burden (TMB) values between inflamed subtype (NIS) and inflamed subtype (IS) from TCGA-DLBCL. Supplementary Figure 3. Boxplot distribution of expression data of PD-1 between inflamed subtype (NIS) and inflamed subtype (IS) from six data sets. Supplementary Figure 4. Boxplot distribution of expression data of PD-L1 between inflamed subtype (NIS) and inflamed subtype (IS) from six data sets. Supplementary Figure 5. Identification of differentially expressed genes (DEGs) between inflamed subtype (NIS) and inflamed subtype (IS) by expression profiling of TCGA-DLBCL, GSE21846, GSE32918, GSE11318, and GSE23501 data sets. The significantly upregulated and downregulated DEGs were shown in a heatmap by log2FoldChange values. Red represents higher expression and green represents lower expression in IS samples. Supplementary Figure 6. Boxplot distribution of expression data of 12 selected genes and between inflamed subtype (NIS) and inflamed subtype (IS) from the testing set (GSE10846). Supplementary Figure 7. Analysis of the relationship between 12 selected genes and diffuse large B-cell lymphoma (DLBCL) overall survival prognosis based on the Kaplan–Meier plotter in the testing set (GSE10846). (a–f) Kaplan–Meier plots of survival analysis of 6 upregulated genes. (g–l) Kaplan–Meier plots of survival anal [file 3168172.f1.zip › supplementary Figure 2.pdf]

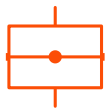

NIS

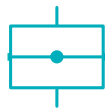

IS

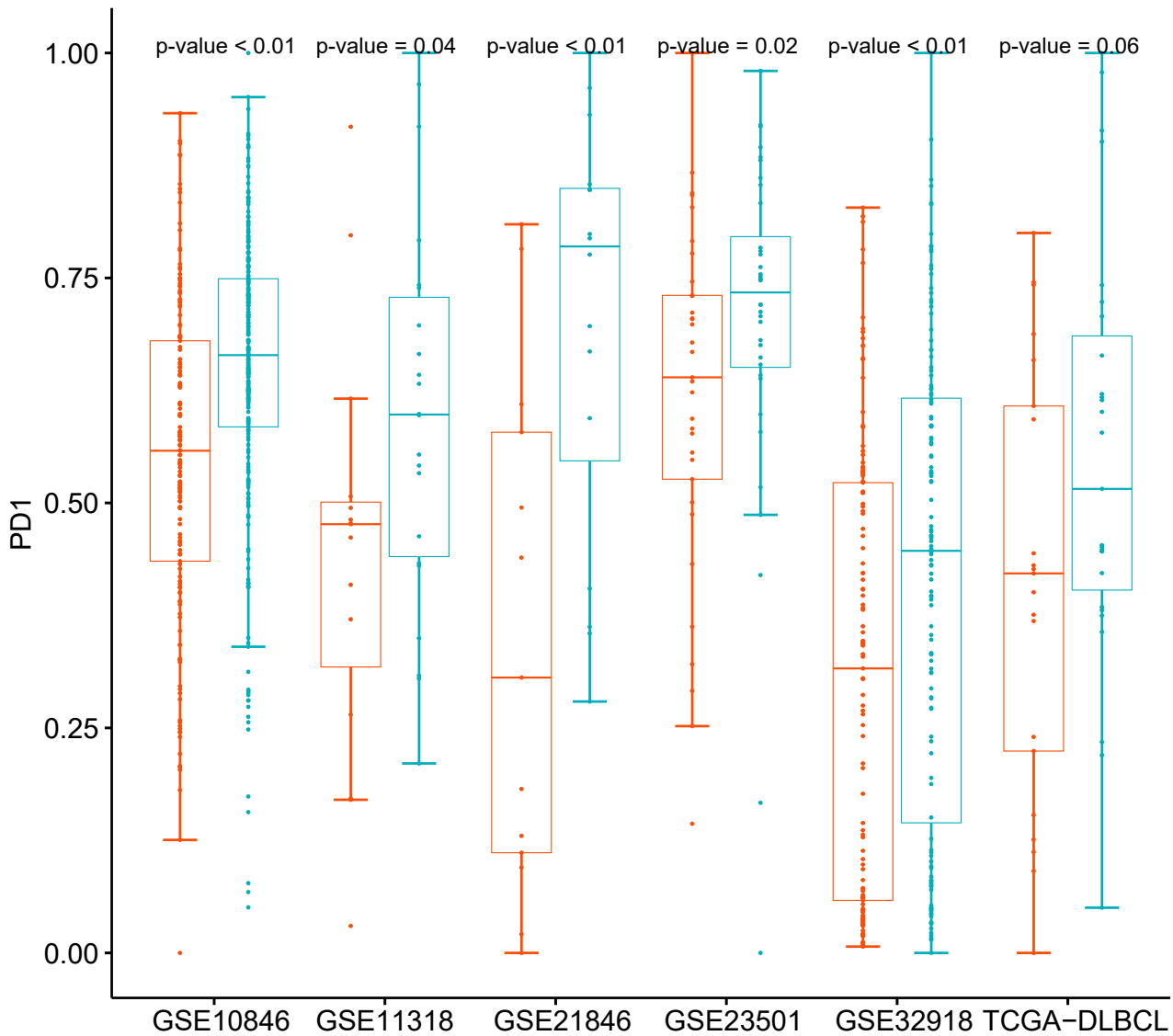

Supplement: Supplementary Materials — Supplementary Figure 1. The testing cohort showed heterogeneity of immune infiltration among the NIS and IS. (a) Higher abundance of immune cells such as activated CD4+ T cells, activated CD8+ T cells, and natural killer cells were observed in the IS, while higher abundance of B cell types containing activated B cells (p value = 0.02), immature B cells (p value = 0.65), and memory B cells (p value = 0.39) were higher in the NIS. (b) For most types of the immune process, they were higher in the IS. But the B cell receptor signaling process was not significantly different between the two subtypes (p value = 0.53). Supplementary Figure 2. Boxplot distribution of tumor mutational burden (TMB) values between inflamed subtype (NIS) and inflamed subtype (IS) from TCGA-DLBCL. Supplementary Figure 3. Boxplot distribution of expression data of PD-1 between inflamed subtype (NIS) and inflamed subtype (IS) from six data sets. Supplementary Figure 4. Boxplot distribution of expression data of PD-L1 between inflamed subtype (NIS) and inflamed subtype (IS) from six data sets. Supplementary Figure 5. Identification of differentially expressed genes (DEGs) between inflamed subtype (NIS) and inflamed subtype (IS) by expression profiling of TCGA-DLBCL, GSE21846, GSE32918, GSE11318, and GSE23501 data sets. The significantly upregulated and downregulated DEGs were shown in a heatmap by log2FoldChange values. Red represents higher expression and green represents lower expression in IS samples. Supplementary Figure 6. Boxplot distribution of expression data of 12 selected genes and between inflamed subtype (NIS) and inflamed subtype (IS) from the testing set (GSE10846). Supplementary Figure 7. Analysis of the relationship between 12 selected genes and diffuse large B-cell lymphoma (DLBCL) overall survival prognosis based on the Kaplan–Meier plotter in the testing set (GSE10846). (a–f) Kaplan–Meier plots of survival analysis of 6 upregulated genes. (g–l) Kaplan–Meier plots of survival anal [file 3168172.f1.zip › supplementary Figure 3.pdf]

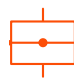

NIS

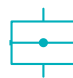

IS

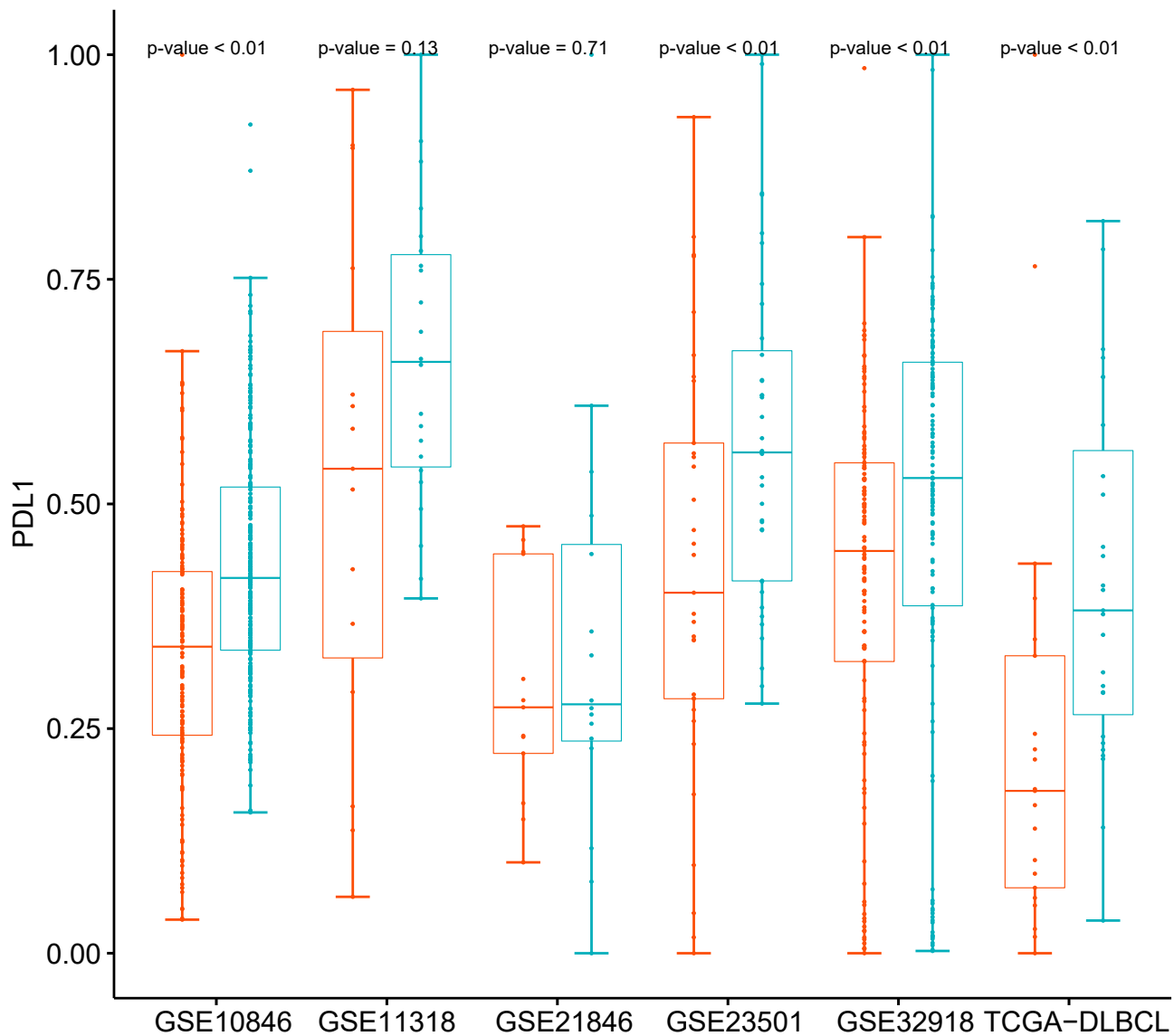

Supplement: Supplementary Materials — Supplementary Figure 1. The testing cohort showed heterogeneity of immune infiltration among the NIS and IS. (a) Higher abundance of immune cells such as activated CD4+ T cells, activated CD8+ T cells, and natural killer cells were observed in the IS, while higher abundance of B cell types containing activated B cells (p value = 0.02), immature B cells (p value = 0.65), and memory B cells (p value = 0.39) were higher in the NIS. (b) For most types of the immune process, they were higher in the IS. But the B cell receptor signaling process was not significantly different between the two subtypes (p value = 0.53). Supplementary Figure 2. Boxplot distribution of tumor mutational burden (TMB) values between inflamed subtype (NIS) and inflamed subtype (IS) from TCGA-DLBCL. Supplementary Figure 3. Boxplot distribution of expression data of PD-1 between inflamed subtype (NIS) and inflamed subtype (IS) from six data sets. Supplementary Figure 4. Boxplot distribution of expression data of PD-L1 between inflamed subtype (NIS) and inflamed subtype (IS) from six data sets. Supplementary Figure 5. Identification of differentially expressed genes (DEGs) between inflamed subtype (NIS) and inflamed subtype (IS) by expression profiling of TCGA-DLBCL, GSE21846, GSE32918, GSE11318, and GSE23501 data sets. The significantly upregulated and downregulated DEGs were shown in a heatmap by log2FoldChange values. Red represents higher expression and green represents lower expression in IS samples. Supplementary Figure 6. Boxplot distribution of expression data of 12 selected genes and between inflamed subtype (NIS) and inflamed subtype (IS) from the testing set (GSE10846). Supplementary Figure 7. Analysis of the relationship between 12 selected genes and diffuse large B-cell lymphoma (DLBCL) overall survival prognosis based on the Kaplan–Meier plotter in the testing set (GSE10846). (a–f) Kaplan–Meier plots of survival analysis of 6 upregulated genes. (g–l) Kaplan–Meier plots of survival anal [file 3168172.f1.zip › supplementary Figure 4.pdf]

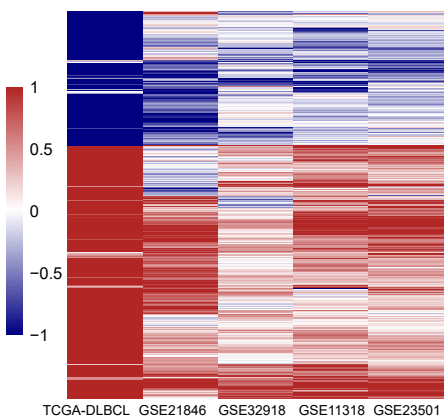

Supplement: Supplementary Materials — Supplementary Figure 1. The testing cohort showed heterogeneity of immune infiltration among the NIS and IS. (a) Higher abundance of immune cells such as activated CD4+ T cells, activated CD8+ T cells, and natural killer cells were observed in the IS, while higher abundance of B cell types containing activated B cells (p value = 0.02), immature B cells (p value = 0.65), and memory B cells (p value = 0.39) were higher in the NIS. (b) For most types of the immune process, they were higher in the IS. But the B cell receptor signaling process was not significantly different between the two subtypes (p value = 0.53). Supplementary Figure 2. Boxplot distribution of tumor mutational burden (TMB) values between inflamed subtype (NIS) and inflamed subtype (IS) from TCGA-DLBCL. Supplementary Figure 3. Boxplot distribution of expression data of PD-1 between inflamed subtype (NIS) and inflamed subtype (IS) from six data sets. Supplementary Figure 4. Boxplot distribution of expression data of PD-L1 between inflamed subtype (NIS) and inflamed subtype (IS) from six data sets. Supplementary Figure 5. Identification of differentially expressed genes (DEGs) between inflamed subtype (NIS) and inflamed subtype (IS) by expression profiling of TCGA-DLBCL, GSE21846, GSE32918, GSE11318, and GSE23501 data sets. The significantly upregulated and downregulated DEGs were shown in a heatmap by log2FoldChange values. Red represents higher expression and green represents lower expression in IS samples. Supplementary Figure 6. Boxplot distribution of expression data of 12 selected genes and between inflamed subtype (NIS) and inflamed subtype (IS) from the testing set (GSE10846). Supplementary Figure 7. Analysis of the relationship between 12 selected genes and diffuse large B-cell lymphoma (DLBCL) overall survival prognosis based on the Kaplan–Meier plotter in the testing set (GSE10846). (a–f) Kaplan–Meier plots of survival analysis of 6 upregulated genes. (g–l) Kaplan–Meier plots of survival anal [file 3168172.f1.zip › supplementary Figure 5.pdf]

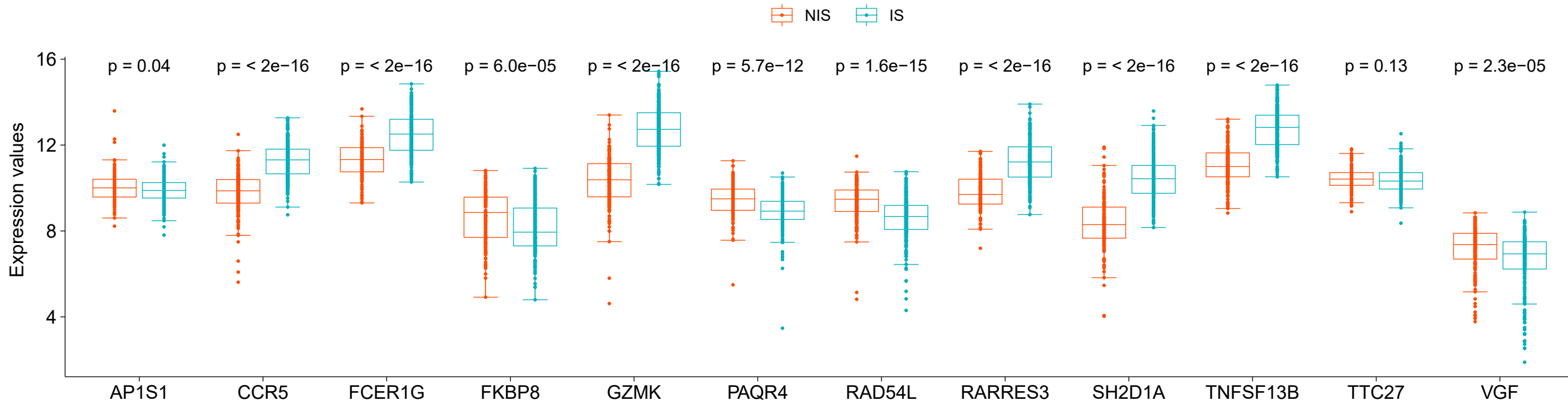

Supplement: Supplementary Materials — Supplementary Figure 1. The testing cohort showed heterogeneity of immune infiltration among the NIS and IS. (a) Higher abundance of immune cells such as activated CD4+ T cells, activated CD8+ T cells, and natural killer cells were observed in the IS, while higher abundance of B cell types containing activated B cells (p value = 0.02), immature B cells (p value = 0.65), and memory B cells (p value = 0.39) were higher in the NIS. (b) For most types of the immune process, they were higher in the IS. But the B cell receptor signaling process was not significantly different between the two subtypes (p value = 0.53). Supplementary Figure 2. Boxplot distribution of tumor mutational burden (TMB) values between inflamed subtype (NIS) and inflamed subtype (IS) from TCGA-DLBCL. Supplementary Figure 3. Boxplot distribution of expression data of PD-1 between inflamed subtype (NIS) and inflamed subtype (IS) from six data sets. Supplementary Figure 4. Boxplot distribution of expression data of PD-L1 between inflamed subtype (NIS) and inflamed subtype (IS) from six data sets. Supplementary Figure 5. Identification of differentially expressed genes (DEGs) between inflamed subtype (NIS) and inflamed subtype (IS) by expression profiling of TCGA-DLBCL, GSE21846, GSE32918, GSE11318, and GSE23501 data sets. The significantly upregulated and downregulated DEGs were shown in a heatmap by log2FoldChange values. Red represents higher expression and green represents lower expression in IS samples. Supplementary Figure 6. Boxplot distribution of expression data of 12 selected genes and between inflamed subtype (NIS) and inflamed subtype (IS) from the testing set (GSE10846). Supplementary Figure 7. Analysis of the relationship between 12 selected genes and diffuse large B-cell lymphoma (DLBCL) overall survival prognosis based on the Kaplan–Meier plotter in the testing set (GSE10846). (a–f) Kaplan–Meier plots of survival analysis of 6 upregulated genes. (g–l) Kaplan–Meier plots of survival anal [file 3168172.f1.zip › supplementary Figure 6.pdf]

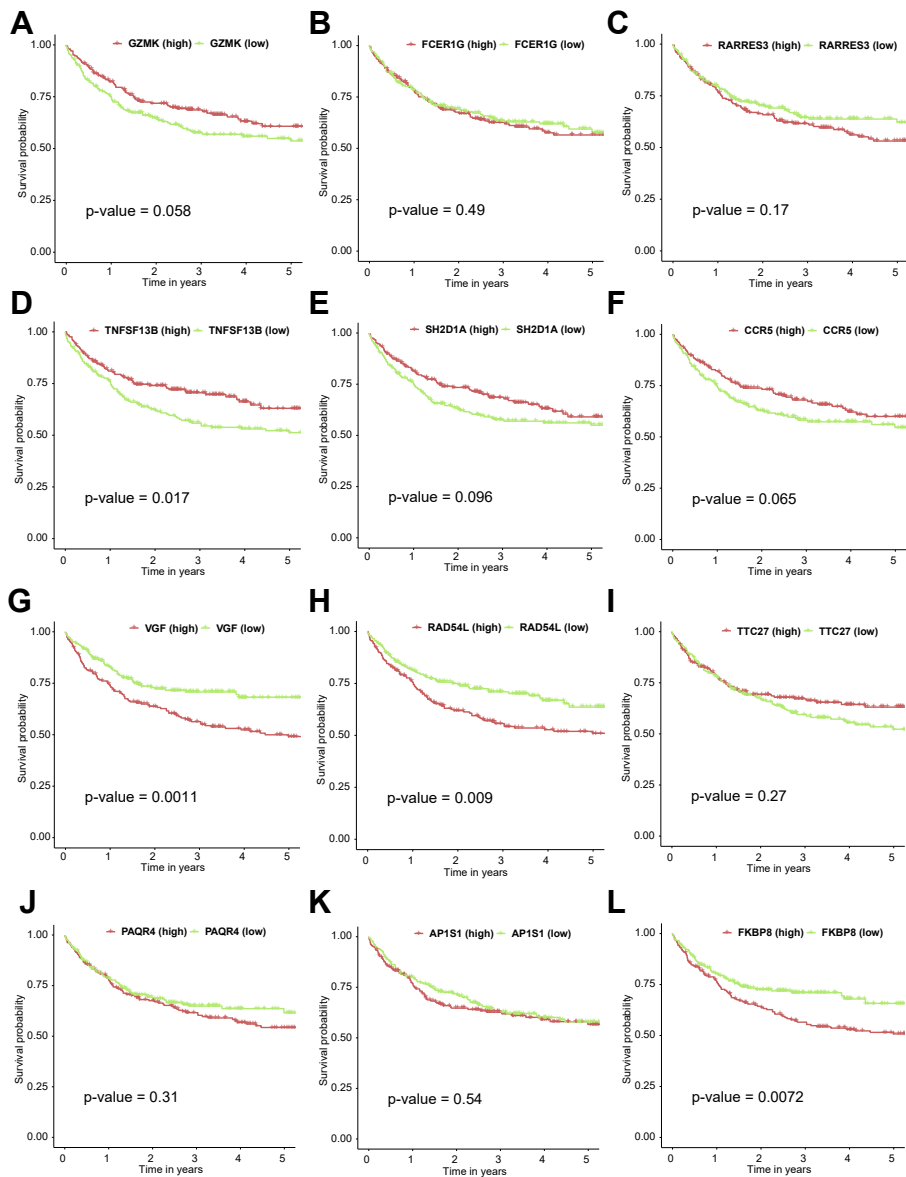

Supplement: Supplementary Materials — Supplementary Figure 1. The testing cohort showed heterogeneity of immune infiltration among the NIS and IS. (a) Higher abundance of immune cells such as activated CD4+ T cells, activated CD8+ T cells, and natural killer cells were observed in the IS, while higher abundance of B cell types containing activated B cells (p value = 0.02), immature B cells (p value = 0.65), and memory B cells (p value = 0.39) were higher in the NIS. (b) For most types of the immune process, they were higher in the IS. But the B cell receptor signaling process was not significantly different between the two subtypes (p value = 0.53). Supplementary Figure 2. Boxplot distribution of tumor mutational burden (TMB) values between inflamed subtype (NIS) and inflamed subtype (IS) from TCGA-DLBCL. Supplementary Figure 3. Boxplot distribution of expression data of PD-1 between inflamed subtype (NIS) and inflamed subtype (IS) from six data sets. Supplementary Figure 4. Boxplot distribution of expression data of PD-L1 between inflamed subtype (NIS) and inflamed subtype (IS) from six data sets. Supplementary Figure 5. Identification of differentially expressed genes (DEGs) between inflamed subtype (NIS) and inflamed subtype (IS) by expression profiling of TCGA-DLBCL, GSE21846, GSE32918, GSE11318, and GSE23501 data sets. The significantly upregulated and downregulated DEGs were shown in a heatmap by log2FoldChange values. Red represents higher expression and green represents lower expression in IS samples. Supplementary Figure 6. Boxplot distribution of expression data of 12 selected genes and between inflamed subtype (NIS) and inflamed subtype (IS) from the testing set (GSE10846). Supplementary Figure 7. Analysis of the relationship between 12 selected genes and diffuse large B-cell lymphoma (DLBCL) overall survival prognosis based on the Kaplan–Meier plotter in the testing set (GSE10846). (a–f) Kaplan–Meier plots of survival analysis of 6 upregulated genes. (g–l) Kaplan–Meier plots of survival anal [file 3168172.f1.zip › supplementary Figure 7.pdf]

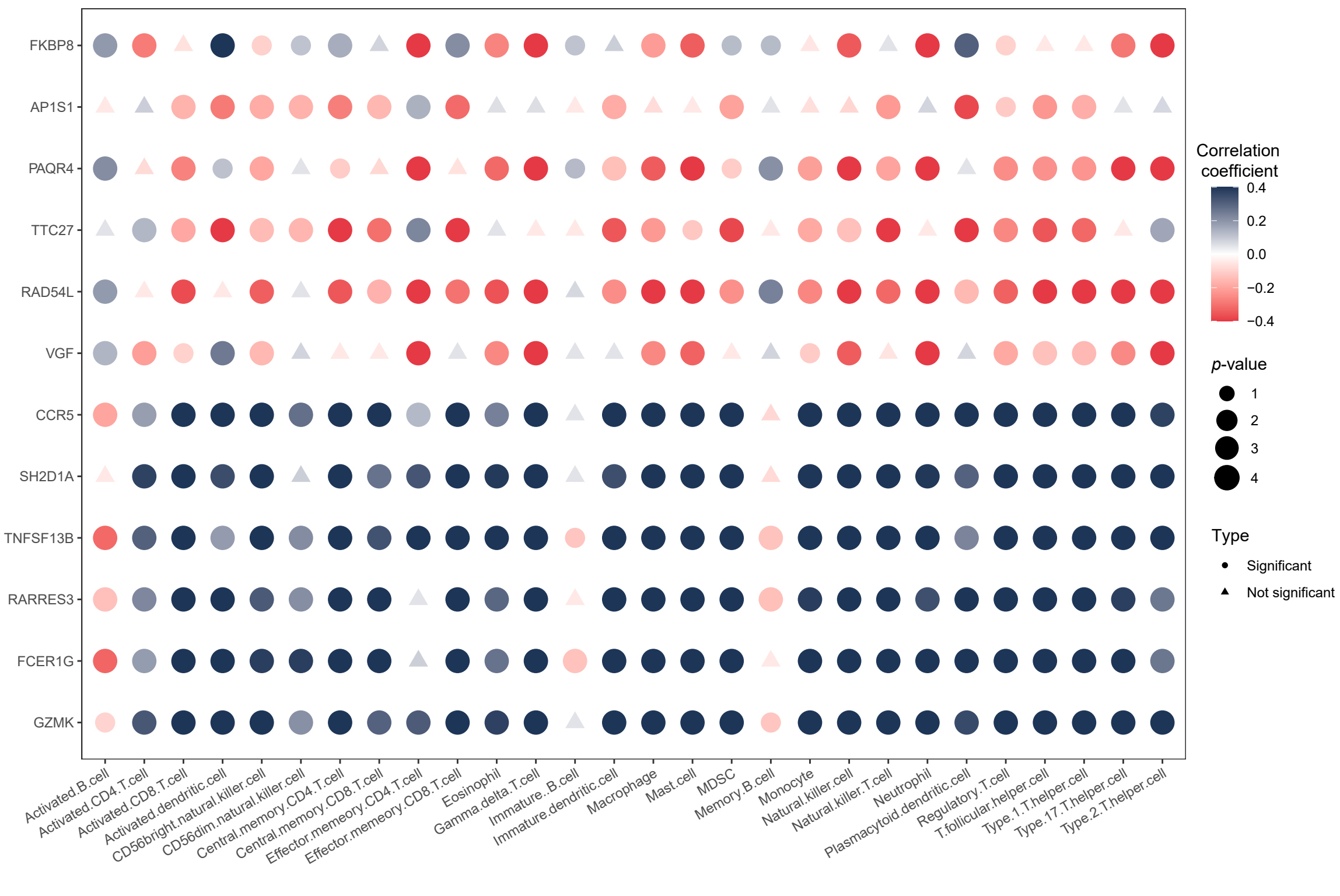

Supplement: Supplementary Materials — Supplementary Figure 1. The testing cohort showed heterogeneity of immune infiltration among the NIS and IS. (a) Higher abundance of immune cells such as activated CD4+ T cells, activated CD8+ T cells, and natural killer cells were observed in the IS, while higher abundance of B cell types containing activated B cells (p value = 0.02), immature B cells (p value = 0.65), and memory B cells (p value = 0.39) were higher in the NIS. (b) For most types of the immune process, they were higher in the IS. But the B cell receptor signaling process was not significantly different between the two subtypes (p value = 0.53). Supplementary Figure 2. Boxplot distribution of tumor mutational burden (TMB) values between inflamed subtype (NIS) and inflamed subtype (IS) from TCGA-DLBCL. Supplementary Figure 3. Boxplot distribution of expression data of PD-1 between inflamed subtype (NIS) and inflamed subtype (IS) from six data sets. Supplementary Figure 4. Boxplot distribution of expression data of PD-L1 between inflamed subtype (NIS) and inflamed subtype (IS) from six data sets. Supplementary Figure 5. Identification of differentially expressed genes (DEGs) between inflamed subtype (NIS) and inflamed subtype (IS) by expression profiling of TCGA-DLBCL, GSE21846, GSE32918, GSE11318, and GSE23501 data sets. The significantly upregulated and downregulated DEGs were shown in a heatmap by log2FoldChange values. Red represents higher expression and green represents lower expression in IS samples. Supplementary Figure 6. Boxplot distribution of expression data of 12 selected genes and between inflamed subtype (NIS) and inflamed subtype (IS) from the testing set (GSE10846). Supplementary Figure 7. Analysis of the relationship between 12 selected genes and diffuse large B-cell lymphoma (DLBCL) overall survival prognosis based on the Kaplan–Meier plotter in the testing set (GSE10846). (a–f) Kaplan–Meier plots of survival analysis of 6 upregulated genes. (g–l) Kaplan–Meier plots of survival anal [file 3168172.f1.zip › supplementary Figure 8.pdf]
